# The E3 ubiquitin ligase UBE3A is an integral component of the molecular circadian clock through regulating the BMAL1 transcription factor

**DOI:** 10.1093/nar/gku225

**Published:** 2014-04-11

**Authors:** Nicole C. Gossan, Feng Zhang, Baoqiang Guo, Ding Jin, Hikari Yoshitane, Aiyu Yao, Nick Glossop, Yong Q. Zhang, Yoshitaka Fukada, Qing-Jun Meng

**Affiliations:** 1 Faculty of Life Sciences, University of Manchester, Oxford Road, Manchester, M13 9PT, UK; 2 Department of Biophysics and Biochemistry, Graduate School of Science, University of Tokyo, Tokyo 113-0033, Japan; 3 Institute of Genetics and Developmental Biology, Chinese Academy of Sciences, Beijing 100101, China

## Abstract

Post-translational modifications (such as ubiquitination) of clock proteins are critical in maintaining the precision and robustness of the evolutionarily conserved circadian clock. Ubiquitination of the core clock transcription factor BMAL1 (brain and muscle Arnt-like 1) has recently been reported. However, it remains unknown whether BMAL1 ubiquitination affects circadian pacemaking and what ubiquitin ligase(s) is involved. Here, we show that activating UBE3A (by expressing viral oncogenes E6/E7) disrupts circadian oscillations in mouse embryonic fibroblasts, measured using PER2::Luc dynamics, and rhythms in endogenous messenger ribonucleic acid and protein levels of BMAL1. Over-expression of E6/E7 reduced the level of BMAL1, increasing its ubiquitination and proteasomal degradation. UBE3A could bind to and degrade BMAL1 in a ubiquitin ligase-dependent manner. This occurred both in the presence and absence of E6/E7. We provide *in vitro* (knockdown/over-expression in mammalian cells) and *in vivo* (genetic manipulation in *Drosophila*) evidence for an endogenous role of UBE3A in regulating circadian dynamics and rhythmic locomotor behaviour. Together, our data reveal an essential and conserved role of UBE3A in the regulation of the circadian system in mammals and flies and identify a novel mechanistic link between oncogene E6/E7-mediated cell transformation and circadian (BMAL1) disruption.

## INTRODUCTION

Circadian clocks drive ∼24-h rhythms in gene expression, physiology and behaviour and are strikingly well conserved in flies and mammals (1,2). At the molecular level, circadian oscillations rely on transcriptional/translational feedback loops. In mammals, the circadian transcription factors BMAL1 (brain and muscle Arnt-like 1) and CLOCK activate transcription of two repressors PERIODS (PERs) and CRYPTOCHROMES (CRYs) in turn feedback to periodically suppress their own transcription (1,3).

Post-translational modifications modulate protein stability to ensure robust 24-h oscillations. Timely degradation of the circadian clock proteins (such as PERs, CRYs and REV-ERBα) has been shown to be essential for circadian timing (4–8). Degradation by the 26S proteasome requires the transfer of ubiquitin moieties to substrate proteins, the final step of which requires a substrate specific E3 ubiquitin protein ligase. It is known that BMAL1 is targeted for degradation by ubiquitination (9). However, little is known about the ubiquitin ligase(s) targeting BMAL1and the potential significance of this to circadian pacemaking.

Of the ubiquitin ligases, UBE3A (ubiquitin protein ligase E3A, also known as E6-AP, E6-associated protein) is unique in that it can be activated by the E6 oncoprotein from high risk human papillomaviruses (HPV16/18), in addition to its normal physiological regulators. HPV infections underlie several human cancers and are responsible for almost all cervical cancers (10,11). During tumorigenesis, the E6/UBE3A complex targets p53 for ubiquitination and degradation (12), a major tumour suppressor. However, additional E6 targets have been identified that often contribute to tumour formation (13,14). Core clock proteins interact with components of the cell cycle to rhythmically control cell-cycle genes, such as *Wee1, Cyclin D1, C-myc* and *p21(Waf1/Cip1)* (15–21), and PERIODs and BMAL1 have been described as tumour suppressors (16,20,21). To date, a direct link between cell transformation and circadian clock disruption has not been established. Here, we show that E6/E7-mediated cell transformation disrupts molecular clock rhythms through increasing UBE3A-dependent degradation of BMAL1. Further, we provide experimental evidence implicating UBE3A as an endogenous key regulator of the circadian pacemaking in mammals and flies, revealing the physiological role of UBE3A in the biological timing system.

## MATERIALS AND METHODS

### Mice

PER2::Luc mice were from Prof. J Takahashi (22). All animal work was carried out in accordance with the 1986 Home Office Animal Procedures Act (UK) and following local ethical review.

### Antibodies and reagents

The following antibodies were used in this study, BMAL1 (mouse monoclonal, 23), ubiquitin (Abcam, rabbit polyclonal), REV-ERBα (kind gift from Dr J. Ripperger), UBE3A (Cell Signalling, rabbit monoclonal), total GSK3β and p-GSK3β (Cell Signalling, rabbit polyclonal), Myc and tubulin (Sigma, monoclonal), Human influenza hemagglutinin (HA) (Santa Cruz, mouse monoclonal), Histidine tag (HIS) (Santa Cruz, mouse monoclonal), V5 (Invitrogen, mouse monoclonal), p53 (Merck Millipore, mouse monoclonal) and green fluorescent protein (GFP) (Santa Cruz, rabbit polyclonal). MG132, dexamethasone, forskolin and cycloheximide were purchased from Sigma.

### Plasmids

mCLOCK and hBMAL1 expression plasmids were kind gifts from Dr Kazuhiro Yagita (Kyoto Prefectural University of Medicine, Japan). *mAVP*::luc plasmid and luc assay were described previously (23,24). Full-length complementary deoxyribonucleic acid (cDNA) encoding V5-BMAL1 was a kind gift from Dr Pat Nolan (MRC Harwell, UK), pCW7-HIS-ubiquitin were described previously(25). HA-UBE3A (Addgene 8648), HA-UBE3A-C833A (Addgene 8649), Myc-UBE3A (Addgene 11468) and E6/E7 (Addgene 8641).

### Mouse embryonic fibroblast (MEF) isolation and transfection

MEFs were isolated from E13.5 wild type (WT) PER2::Luc mice as described (26). MEFs were transfected with E6/E7 construct as described before (27) and stable transfectants confirmed by genotyping and reverse transcriptase-polymerase chain reaction (RT-PCR) of E6/E7 fragments from genomic DNA and cDNA. NIH3T3 cells were purchased from the American Type Culture Collection. Cells were grown in complete Dulbecco's modified Eagle's medium (DMEM; Invitrogen) supplemented with 10% fetal bovine serum (FBS) in a 37°C incubator maintained at 5% CO_2_.

### Soft agar assay

Agarose was prepared in 6-well plates, with a base layer consisting of 1% agar in 1 x DMEM supplemented with 10% FBS and a top layer consisting of 0.35% agar in 1 x DMEM with 10% FBS. Cells were seeded directly into the top agar layer at a density of 5 × 10^3^ cells per well and allowed to grow for approximately 3 weeks prior to imaging under phase contrast.

### Immunocytochemistry of p53

Immunocytochemistry was performed as described (24). Briefly, cells on sterile coverslips were fixed with 4% paraformaldehyde, then blocked in 1% donkey serum before incubation with primary antibody. Cells were then treated with an Alexa-Fluor-conjugated secondary antibody mix. Fluorescence was visualized using a Zeiss Observer D1 AX10-inverted microscope, photographed using an AxioCam ICM1 camera and AxioVision version 4.8.2 software (Carl Zeiss).

### Real-time bioluminescence recording and analysis

Circadian rhythms in bioluminescence from *Per2*::Luc in SW1353 cells or PER2::Luc in MEFs were recorded in real time using photomultiplier tube (PMT) devices (28). Confluent cells were synchronized with 50% horse serum for 2 h, Dex (100 nM), Forskolin (FSK) (10 μM) or temperature cycles (36–38.5°C) as indicated, then transferred to standard recording media as described (29). For shRNA knockdown in NIH3T3 cells with pGL3-*mBmal1*::Luc-Hyg (30), 35-mm dishes of cells were transiently transfected by shRNA vectors. The cells were treated with 100-nM Dex for 2 h and then transferred to standard recording media. Bioluminescence was recorded for 1 min at 9-min intervals for 5–10 days at 37ºC in air with Dish Type Luminescencer, Kronos (ATTO). Raw data of the rhythms were detrended by 24-h moving averages.

### Transfections, immunoblotting and protein stability assay

Whole cell extracts were prepared in lysis buffer containing 1% Triton X-100 as previously described (25). Plasmid transfections for HEK293T or NIH3T3 cells were performed using Polyfect (Qiagen) according to the manufacturer's instructions. To analyse protein stability, the protein synthesis inhibitor cycloheximide (20 μg/ml) was added to cells for the times indicated in the figure legends. Cells were then washed and samples analysed over a 6-h period by western blot.

### Ubiquitination assay

Ubiquitination of BMAL1 was examined by histidine (His) purification method (25). HEK 293T cells were transfected with V5-BMAL1, His-ubiquitin, E6/E7, wild-type or mutant derivatives of UBE3A plasmids in varied combination as detailed in figure legend. After transfection, the cells were left overnight and then treated with MG132 for 6 h, followed by solubilizing the cells with lysis buffer A containing 8-M guanidinium HCl, 100-mM Na2HPO4/NaH2PO4,10-mM Tris-HCl (pH8.0) and 10-mM imidazole. Subsequently, Ni-nitrilotriacetic acid purification of the His-ubiquitin conjugates was performed under denaturing conditions.

### Co-immunoprecipitation (Co-IP) of Myc-UBE3A, HA-UBE3A and V5-BMAL1

Co-IP of over-expressed protein was performed as described before (25). Briefly, HEK 293T cells in 10-cm dishes were transfected with various combinations of tagged plasmids. Overnight after transfection, the cells were solubilized with lysis buffer (150-mM NaCl, 20-mM Tris-HCl, pH7.5, 10% glycerol, 1% Triton X-100, 1-mM phenylmethylsulfonyl fluoride, PhosSTOP and cOmplete ethylenediaminetetraacetic acid-free cocktail protease inhibitors, Roche). After centrifugation, 1-ml supernatants were used for immunoprecipitation by adding 2 μg of V5 or HA antibody as appropriate at 4°C overnight. The following day, Protein G Dynabeads (Invitrogen) were added and incubated for 2 h, followed by four washes with lysis buffer in the presence of protease inhibitors. Precipitated proteins were examined by sodium dodecyl sulphate-polyacrylamide gel electrophoresis (SDS-PAGE) and western blot.

### Gene expression analysis

Ribonucleic acid (RNA) was extracted from cultured fibroblasts using the Qiagen RNeasy purification system. cDNA was prepared using the Superscript II reverse transcriptase (Invitrogen) and analysed for gene expression using quantitative real-time PCR with TaqMan (Applied Biosystems) chemistry (24). Probeset was ordered (pre-validated) from Applied Biosystems (*Bmal1* Mm00500226_m1).

### ShRNA and siRNA transfections

For knockdown of *Ube3a*, shRNA was designed using siDirect (http://sidirect2.rnai.jp/), a web-based software, and the following sequences were used: shUbe3a#1—5′-GGUUU ACUAU GCAAA UGUAG U-3′—and shUbe3a#2—5′-GGAUU AUAUU AUGAC AAUAG A-3′. The oligonucleotides to express the shRNA were inserted into the pBS-mU6 vector, as described previously (30). Small interfering RNA (siRNA) transfections were performed with RNAiMax reverse transfection reagent (Invitrogen). Cells were incubated with 10- or 20-nM SMARTpool siRNA duplexes against human *Ube3a*, or a scrambled duplex (Dharmacon) for 24 h before downstream analysis. For PMT studies, cells were synchronized with 50% horse serum for 2 h before PMT recording. For Western Blotting (WB) analysis of knockdown efficiency, transfected cells were harvested for protein extraction 48 h post transfection.

### *Drosophila* stocks

All *Drosophila* stocks were raised on standard cornmeal food at 25°C. RNAi line *VDRC-45876* was from Vienna Drosophila RNA interference (RNAi) Center. The *UAS-ube3a* transgenic line was constructed by amplifying full-length *Dube3a* from a cDNA pool derived from whole adult flies and cloned into pUAST. Following sequence confirmation the *UAS-Ube3a* was injected into embryos for germline transformation. For specific knockdown of *Dube3a*, *w*;UAS-Gal4;*pdf-*Gal4 flies were crossed with *w*;+;UAS-*Ube3a*-RNAi to generate *w*;UAS-Gal4/+;*pdf-*Gal4/UAS*-Ube3a-RNAi progeny.* For controls each line was, instead, crossed to *w;+;+* to generate *w*;UAS-Gal4/+;*pdf-*Gal4/+ and w;+;UAS-*Ube3a*-RNAi/+. Specific over-expression of DUBE3A was achieved by crossing *w*;UAS-Gal4;*pdf-*Gal4 and *w*;+;UAS-*Dube3a* flies to generate *w*;UAS-Gal4/+;*pdf-*Gal4/UAS*-Dube3a*. Controls *(w*;UAS-Gal4/+;*pdf-*Gal4/+ and w;+;UAS-*Dube3a*/+) were generated by crossing each line to *w;+;+.*

### Western blotting of dUBE3A in flies

For the analysis of protein levels, larval brains were dissected and homogenized in ice-cold lysis buffer [50-mM Tris-HCl (pH 7.4), 150-mM NaCl, 1% NP-40, 0.1% SDS and 1% proteinase inhibitor (Merck)], and subjected to SDS-PAGE according to standard procedures. Western blots were probed with anti-UBE3A (8F7, 1:1000, made by Yong^’^s lab). The specificity of the antibody was confirmed in larval brains from the *Dube3a^35^* null line.

### Circadian activity assays of flies

Circadian locomotor activity of flies was measured as described (31). Briefly, male flies, 1–3 days old, were placed in glass tubes and entrained to a 12-h light–12-h dark cycle (LD) for 5 days and then released into constant dark conditions (DD) for 7–10 days. Locomotor activity was recorded in 30-min bins using DAM2 Drosophila Activity Monitor system (Trikinetics, Waltham). Only flies that survived for 12 days were included in analysis. Data were analysed using El Temps software (Antoni Díez Noguera, University of Barcelona). Periodogram analysis was done using chi-square analysis of LD or DD data, as standard, and categorized as arrhythmic if their rhythm strength (variance%) score was below the rhythmic threshold.

### Statistical analysis

Data were evaluated using Student's *t*-test or one-way ANOVA with Tukey test as indicated. Results are presented as mean ± SEM from at least three independent experiments. Differences were considered significant at the values of *P < 0.05, **P < 0.01 and ***P < 0.001.

## RESULTS

### E6/E7-mediated cell transformation disrupts circadian rhythm and BMAL1 expression in MEFs

**Figure 1.**
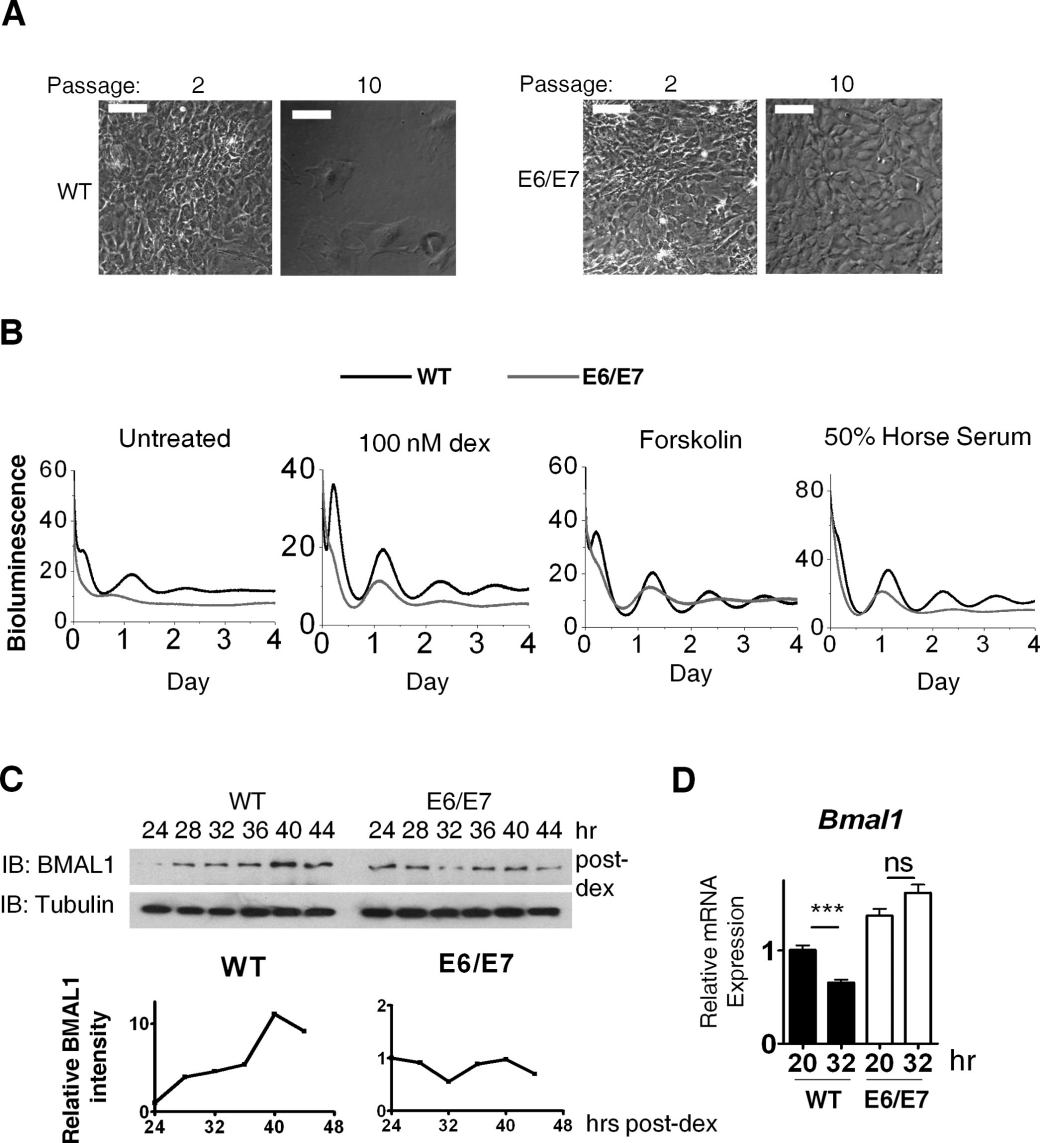
Stable integration and expression of E6/E7 immortalized and transformed MEFs and disrupted the circadian clock. (**A**) Phase contrast micrograph showing E6/E7 cells were capable of growing beyond passage 10 with morphological change while WT primary cells had become senescent. Scale 100 μm. (**B**) Representative PER2::Luc bioluminescence rhythms in WT (*n* = 3) and E6/E7-positive (*n* = 5) cells following three synchronization protocols (dex pulse, forskolin and 50% horse serum). Rhythm dampened rapidly in all cases and period was irregular and significantly longer (28.44 ± 0.77 h compared to 24.83 ± 0.84 h in primary cells; dex synchronized, *P* < 0.05). Y-axis value x1000 = cpm (photon counts per minute). (**C**) Immunoblotting (IB) showing BMAL1 protein rhythm in cells synchronized with dex (100 nM). (**D**) *Bmal1* gene expression (qPCR) in dex-synchronised cells, *n* = 3. ***P < 0.001; ns, not significant.

*In vitro*, over-expression of HPV16 oncogenes E6 and E7 is sufficient to immortalize and transform primary cells (27). In a recent attempt to immortalize MEFs isolated from PER2::Luc clock reporter mice (22), we used E6/E7 oncogenes. Transfection of MEFs with E6/E7 led to successful integration of viral DNA into the host genome (Supplementary Figure S1). While mock transfected MEFs no longer proliferated beyond passage 9, E6/E7 expression led to indefinite proliferation (beyond passage 30; Figure 1A). This was associated with a reduction in p53 protein levels, consistent with E6-mediated degradation as previously reported (Supplementary Figure S2). E6/E7-expressing cells were capable of anchorage-independent growth and colony formation in soft agar, a hallmark of tumorigenic cell lines (Supplementary Figure S3). Thus, stable integration and expression of E6/E7 led to immortalization and transformation of primary MEFs. Surprisingly, using real-time recording, the circadian rhythms in PER2::Luc bioluminescence were severely dampened in transformed MEFs (Figure 1B). This was the case in cells synchronized using various well-accepted protocols (dexamethasone, horse serum, forskolin and 12:12-h temperature cycles; Figure 1B and Supplementary Figure S4). Aberrant rhythms in transformed cells were confirmed by studying BMAL1 protein and messenger RNA (mRNA) expression following clock synchronization (Figure 1C and D).

**Figure 2.**
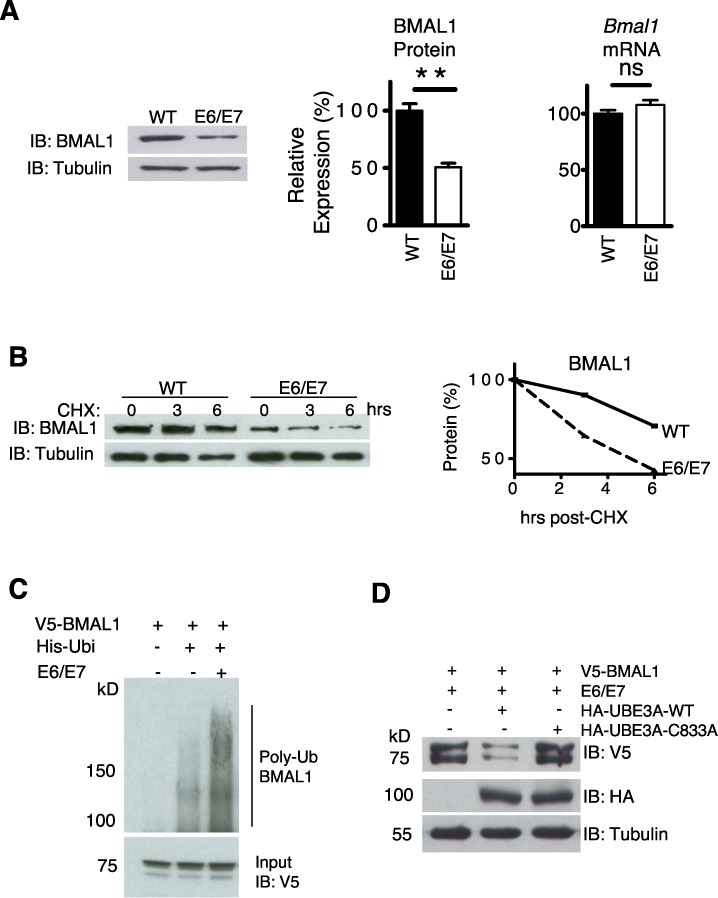
E6/E7 expression enhanced BMAL1 ubiquitination and proteasomal degradation through UBE3A. (**A**) Left panel: IB of steady state BMAL1 in WT and E6/E7 cells. Right panels: integrated density measurement of BMAL1 (*n* = 3, *P* < 0.05) and steady state *Bmal1* gene expression by qPCR (*n* = 3, *P* > 0.05). **P < 0.01, see underlined. (**B**) Top panel: BMAL1 protein level following cycloheximide (20 μg/ml). Bottom panel: protein degradation rate. (**C**) Ubiquitination assay of V5-BMAL1 in HEK 293T cells by nickel affinity pull-down. Total levels of V5-BMAL1 in the input lysates are shown in the panel below. MG132 (5 μM) was applied in all cases. (**D**) V5-BMAL1 levels in HEK 293T cells, transfected with E6/E7, co-transfected with either HA-UBE3A or a dominant negative mutant (C833A). Representative, *n* = 3.

BMAL1 availability has been proposed as ‘rate-limiting’ for the positive arm of the clock and is necessary for robust rhythmicity (32). In unsynchronized cells average BMAL1 levels were significantly lower (*P* < 0.01) in transformed cells than in primary cells. This was not accompanied by a reduction in steady-state *Bmal1* mRNA (Figure 2A, *P* > 0.05). In addition, the levels of REV-ERBα, a key repressor of *Bmal1*, did not significantly change (Supplementary Figure S5A). These results suggest a post-transcriptional mechanism for BMAL1 protein reduction. Indeed, BMAL1 decay rate was faster in transformed cells than in primary cells (Figure 2B) and BMAL1 reduction in transformed cells could be rescued by application of the proteasomal inhibitor MG132 (Supplementary Figure S5B), suggesting the involvement of the proteasomal degradation pathway.

### E6/E7 increases BMAL1 ubiquitination and degradation through UBE3A

BMAL1 is targeted for degradation by ubiquitination (9,33,34), thus we sought to address whether E6/E7 expression affected this. A Ni-NTA His-ubiquitin pull-down assay was used to precipitate ubiquitinated proteins in HEK 293T cells. Immunoblotting revealed that E6/E7 expression enhanced polyubiquitination of V5-BMAL1 (Figure 2C). The final step of ubiquitination requires a substrate-specific E3 ubiquitin protein ligase. Due to the well-documented interaction between E6 and UBE3A (11), we predicted that UBE3A may be involved in E6/E7-mediated degradation of BMAL1. In the presence of E6/E7, over-expression of WT UBE3A, but not a dominant negative mutant (C833A) that lacks ubiquitin ligase activity, caused a reduction in the steady-state level of V5-BMAL1 in HEK 293T cells (Figure 2D). These results suggest that the E6/E7-mediated BMAL1 reduction involves the ubiquitin ligase activity of UBE3A.

### UBE3A co-purifies with BMAL1 in a proteasomal pathway-dependent manner

**Figure 3.**
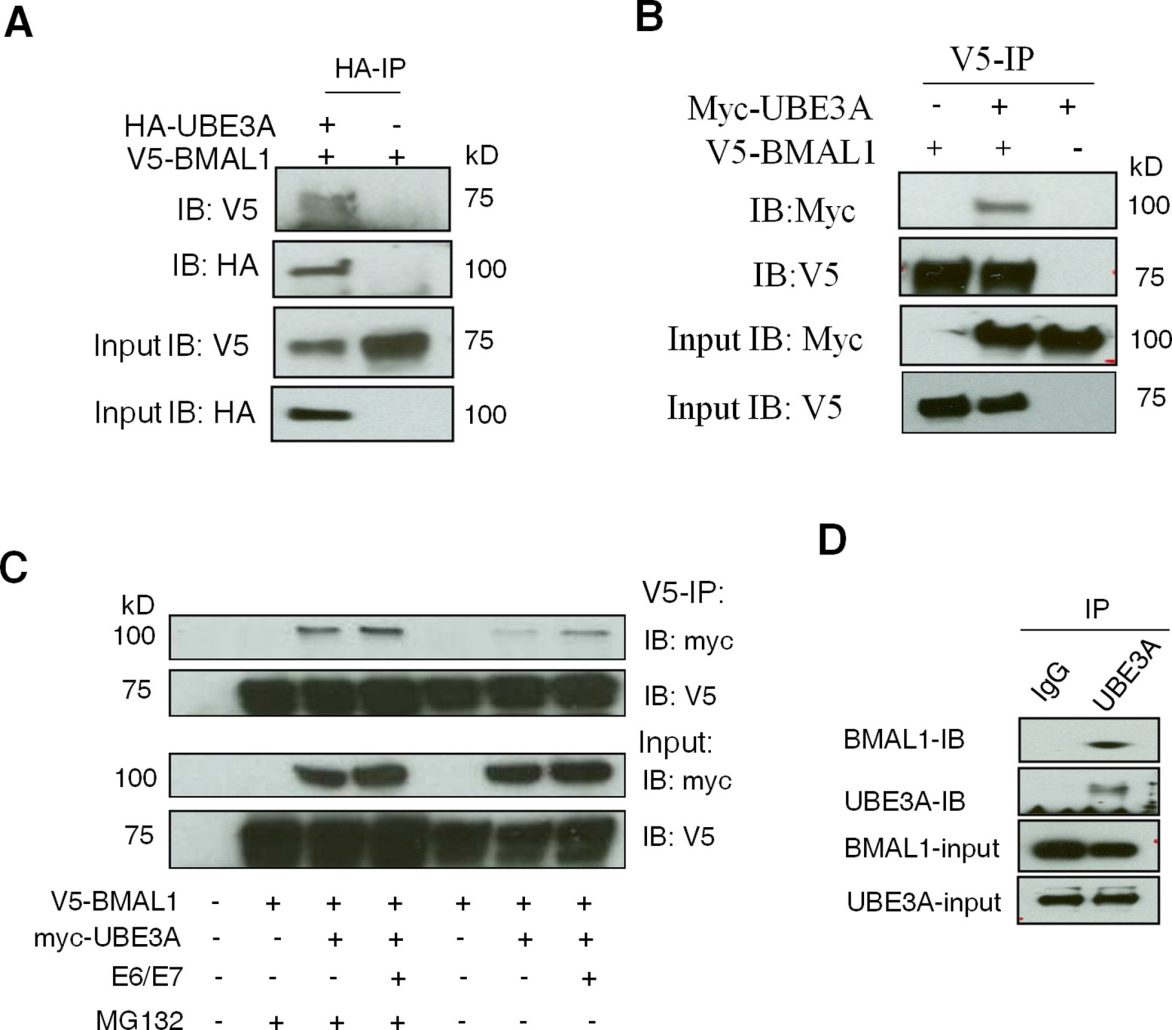
UBE3A interacts with BMAL1 both in the presence and absence of E6/E7. (**A**) Co-IP of HA-UBE3A and V5-BMAL1 in HEK 293T cells. IP was performed using an anti-HA antibody and precipitated V5-BMAL1 or HA-UBE3A was detected by IB using an anti-V5 or anti-HA antibody (top and middle panels). (**B**) V5 Co-IP showing that V5-BMAL1 could pull down Myc-UBE3A. MG132 (5 μM) was applied in all cases. (**C**) Co-IP of V5-BMAL1 and myc-UBE3A in HEK 293T cells in the presence or absence of MG132. IP was performed using an anti-V5 antibody and precipitated myc-UBE3A or V5-BMAL1 was detected by IB (top two panels). (**D**) Endogenous Co-IP between UBE3A and BMAL1 in SW1353 human chondrocytes in the presence of MG132. IP was performed using an anti-UBE3A antibody and the precipitated BMAL1 was detected by IB (top two panels).

To confirm that BMAL1 and UBE3A are capable of interacting with each other in cells, we used Co-IP. In the presence of E6/E7, V5-BMAL1 was pulled down specifically by HA-UBE3A and, reciprocally, myc-UBE3A was pulled down by V5-BMAL1 (Figure 3A and B). Substantially more myc-UBE3A was pulled down when the proteasome was inhibited with MG132, supporting the notion that this interaction leads to proteasomal degradation of BMAL1 (Figure 3C). Importantly, V5-BMAL1 could pull down myc-UBE3A in the presence and absence of E6/E7 (Figure 3C). This is distinct from the E6-dependent interaction between UBE3A and p53 (35), and suggests that UBE3A acts as an endogenous ubiquitin ligase of BMAL1. To prove a physiological interaction between endogenous UBE3A and BMAL1, an endogenous IP was performed in unsynchronized SW1353 cells. Endogenous BMAL1 was pulled down specifically by UBE3A using an anti-UBE3A antibody (Figure 3D). It is known that GSK3β phosphorylates BMAL1, promoting its ubiquitination and degradation (34). However, we found no difference in the level of either total or phosphorylated GSK3β (Ser9) between primary and transformed cells (Supplementary Figure S6).

### UBE3A regulates clock period and amplitude in mammalian cells

**Figure 4.**
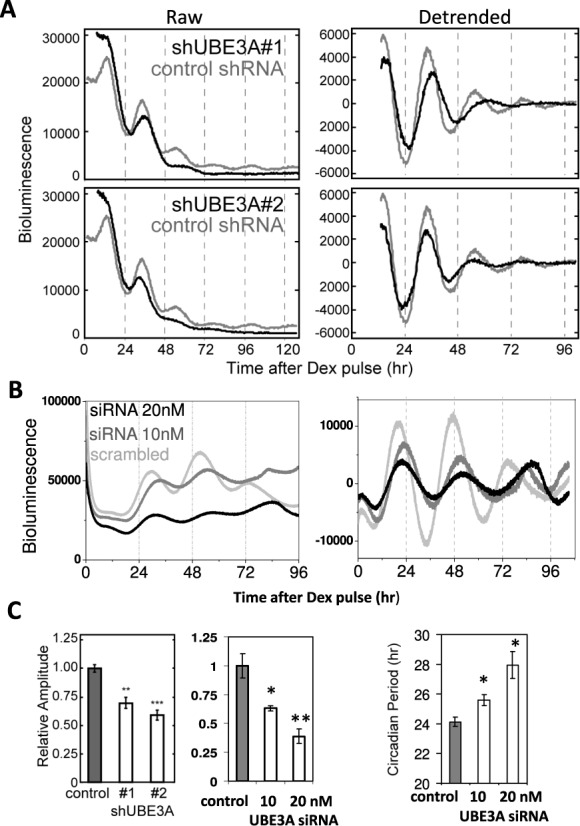
Knockdown of endogenous UBE3A disrupts circadian rhythms in mammalian cells. (**A**) Representative raw (left) and detrended (right) bioluminescence traces of *Bmal1*::Luc NIH3T3 cells treated with shRNA #1 (top) and #2 (bottom) against *Ube3a*. (**B**) Representative traces of *Per2*::luc SW1353 cells treated with human *Ube3a* SiRNA constructs at 10 (mid-grey) and 20 nM (black) doses. Scrambled SiRNA was used as control. (**C**) Left panels: circadian amplitude analysis (*n* = 3, shRNA #1 *P* < 0.01; shRNA #2 *P* < 0.001; RNAi 10 nM, *P* < 0.05; RNAi 20 nM, *P* < 0.01). Right panel: circadian period analysis (*n* = 3, t-tests: RNAi 10 nM, *P* < 0.05; RNAi 20 nM, *P* < 0.05). Note: the rapid dampening of circadian oscillation in shRNA-treated NIH3T3 cells prevented proper evaluation of periodicity. *P < 0.05; **P < 0.01; ***P < 0.001.

Because BMAL1 is essential for rhythmic physiology and UBE3A interacted with BMAL1 under physiological conditions independently of E6/E7, we hypothesized that UBE3A-mediated BMAL1 degradation could be important for endogenous circadian timekeeping. To test this, we used shRNAs to knockdown *Ube3a* in NIH3T3 mouse fibroblasts containing a *Bmal1*::Luc reporter. Knockdown of *Ube3a* led to a significant reduction in the amplitude of *Bmal1*::Luc oscillations (*P* < 0.01 or 0.001; Figure 4A and C and Supplementary Figure S7A). Furthermore, knockdown of human *Ube3a* in a *Per2::*Luc SW1353 human cell line (24) led to unstable clock oscillations, with an initial period lengthening (by up to 4.5 h; *P* < 0.05) and amplitude reduction (∼50%; *P* < 0.05 for 10 nM and *P* < 0.01 for 20-nM siRNA), followed by a loss of (or irregular) rhythmicity (Figure 4B and C). Consistent with a role of UBE3A in regulating BMAL1, we observed increased BMAL1 levels following RNAi treatments against *Ube3a* (Supplementary Figure S7B). Ubiquitination of BMAL1 has been shown to be essential for its transactivational activity (33). To establish a role of endogenous UBE3A in regulating BMAL1 function, we performed transactivation assay in HEK 293 cells using a *mAVP*::luc promoter reporter (23). The ability of BMAL1/CLOCK to activate *mAVP*::luc was significantly reduced by knockdown of *Ube3a* (Supplementary Figure S8), supporting UBE3A as an endogenous regulator of the core clock complex. Taken together, our results demonstrate that loss-of-function of UBE3A in cells dampens circadian amplitude and lengthens clock period, indicating UBE3A as an endogenous regulator of circadian pacemaking in mammalian cells, at least partly through regulating the core clock transcription factor BMAL1.

### Essential role of dUBE3A in controlling rhythmic fly behaviour

**Figure 5.**
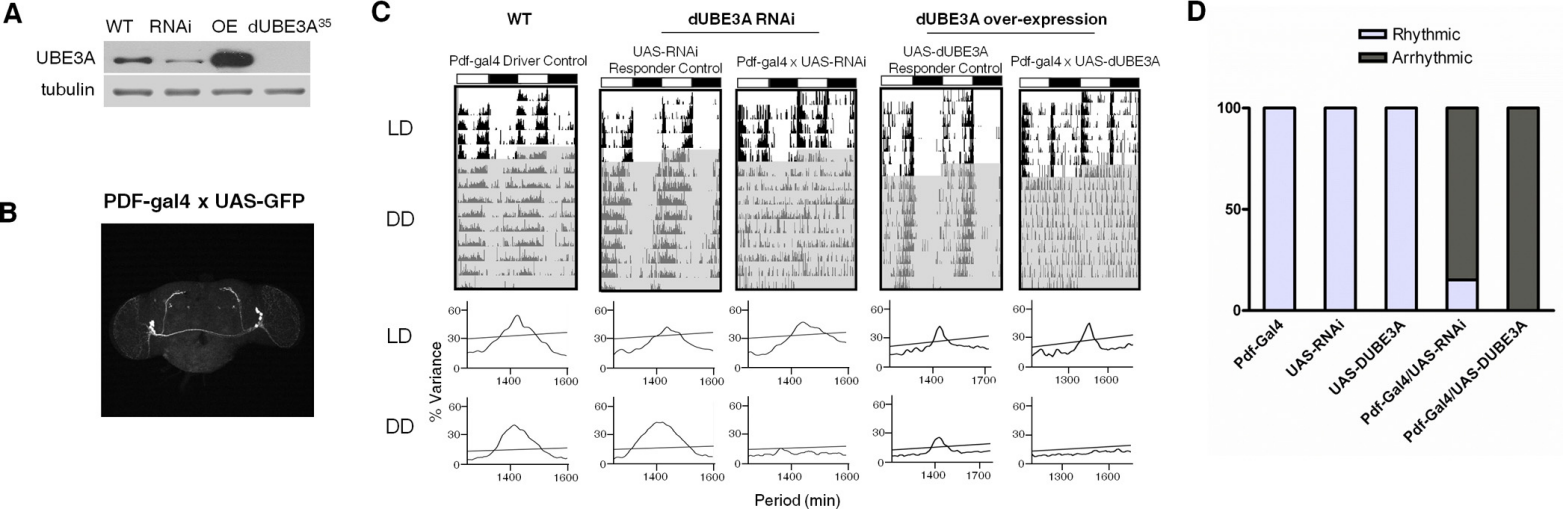
*Drosophila* dUBE3A is necessary for circadian rhythmic behaviour in central clock neurons. (**A**) IB of total protein from L3 larvae brain, showing complete lack of protein in the *dUBE3A*-null (*DUBE3A*^35^), efficiency of *dUBE3A*-RNAi knockdown (RNAi) and over-expression (OE) by the pan-neuronal driver *elav*-Gal4. Two independent blots showed similar results. (**B**) Expression of GFP driven by the *pdf*-Gal4 driver, showing restriction to the ventrolateral neuron (LN_v_) subgroups of central clock cells in the fly brain that coordinate daily sleep-wake cycles. (**C**) Upper panels: representative double-plotted actograms for locomotor activity data in flies with Pigment dispersing factor (PDF) neuron-specific *dUBE3A* RNAi or *dUBE3A* over-expression, along with relevant controls. LD: white background; DD: grey background. Light and dark bars at the top indicate day (ZT0–12) and night (ZT12–24) during LD, or, subjective day (CT0–12)/subjective night (CT12–24) for DD, respectively. Lower panels: representative periodograms during LD and DD for each genotype. (**D**) Relative percentage of rhythmic flies for each genotype; *n* = 10–33.

To determine the importance of UBE3A in driving rhythmic behaviour, we used *Drosophila melanogaster*, which shares high homology with mammals in both the *Ube3a* gene and in molecular clock mechanisms (2,36). We first confirmed effective reduction/increase of average dUBE3A protein levels in all neurons by driving *dUbe3a* RNAi (VDRC-45876) or *dUbe3a*-WT (i.e. over-expression; OE) pan-neuronally (Figure 5A). Next, we used the *pdf*>Gal4 driver to express *dUbe3a* RNAi and WT exclusively in the LN_v_ neurons that coordinate locomotor behaviour (Figure 5B). Both the *pdf*>*dUbe3a-*RNAi and *pdf*>dUBE3A-OE lines exhibited normal behaviour under a 12:12-h light–dark (LD) cycle (Figure 5C and D). The *pdf*>*dUbe3a-*RNAi line gradually lost rhythmicity in DD, with 11/14 flies becoming arrhythmic in DD (Figure 5C and D and Table 1). Surprisingly, the *pdf*>dUBE3A-OE exhibited a similar and more pronounced arrhythmic phenotype, with 100% of flies showing an immediate loss of rhythmicity in DD (Figure 5C and D and Table 1). Taken together, our results indicate that tight control of dUBE3A levels in central clock neurons of flies is critical for the regulation of locomotion rhythms.

**Table 1.** The circadian phenotype of transgenic fly lines expressing *dUbe3a* RNAi (light grey) and over-expressing dUBE3A (dark grey)

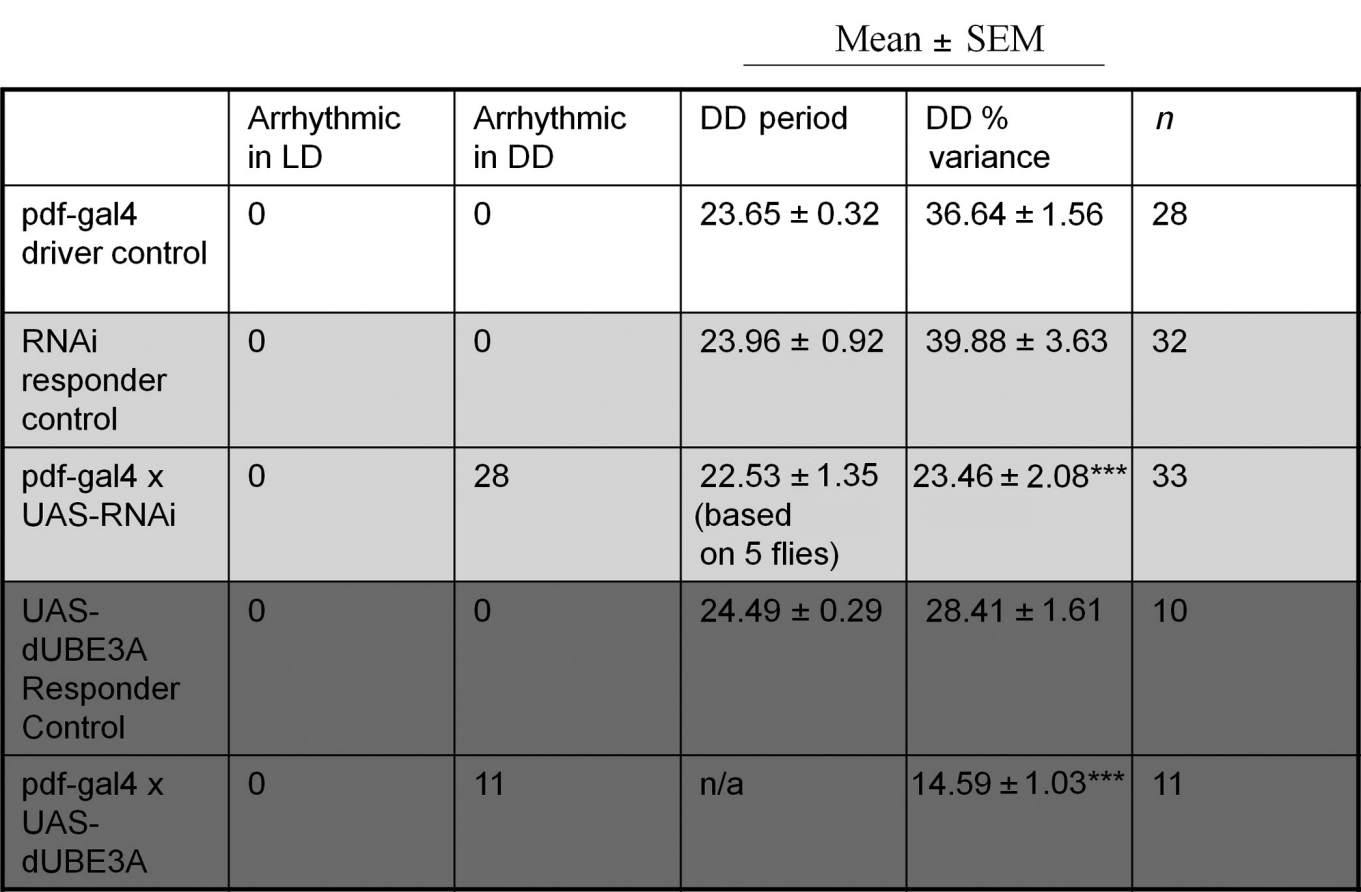					

***P < 0.001.

Period, h; % variance denotes rhythm strength.

## DISCUSSION

Timely degradation of the clock proteins through the ubiquitin-proteasome pathway is critical for the precision and robustness of the circadian clock. In contrast to the extensive studies on the degradation pathways of clock repressors (PERs, CRYs and REV-ERBα) (4–7), the degradation mechanisms of positive clock proteins (such as BMAL1) are poorly understood. In a recent attempt to immortalize MEFs isolated from PER2::Luc reporter mice, we used E6/E7 oncogenes. Unexpectedly, we observed profound disruption of clock gene dynamics in E6/E7-transformed cells. Further investigation identified UBE3A as an endogenous regulator of BMAL1 ubiquitination and degradation, controlling molecular and behavioural rhythmicity in mammalian cells and in flies.

Consistent with the near ubiquitous expression of circadian clocks, UBE3A is widely expressed in multiple mouse tissues, including the central pacemaker superachiasmatic nucleus (SCN) (37 and Supplementary Figure S9). Both over-expression and knockdown of UBE3A in fly central clock neurons resulted in a pronounced circadian phenotype, indicating that precise control of its activity is vital for circadian pacemaking across divergent species. It has been reported that flies carrying a global null mutation of *dUbe3a* demonstrate circadian deficits in locomotor rhythms (36). The clock-neuron-specific manipulations presented here provide unequivocal evidence that dUBE3A is an important regulator of the *Drosophila* central clock and suggests a conserved role of UBE3A in circadian timekeeping in flies and mammals. It is possible that dUBE3A may target CYCLE, the fly orthologue of BMAL1. However, the lack of a commercially available antibody for CYCLE prevented us from testing this possibility. Of note is that UBE3A plays a causal role in a human neuro-genetic disease, Angelman syndrome (38,39). This neurological disorder is characterized by severe intellectual and developmental disability, seizures and sleep disturbance. It is unknown whether the dis-regulation of the UBE3A-mediated circadian rhythms might contribute to some of the symptoms of this human disorder.

BMAL1 ubiquitination has been suggested as a possible global mechanism of clock entrainment (9,33,34). However, our study is the first to report a ubiquitin ligase targeting BMAL1. Interestingly, a recent study revealed UBE3A as a light responsive protein in the mouse SCN (36). It remains to be seen whether UBE3A is involved in light or food-resetting of the circadian clocks. The core clock proteins PERIODs (and BMAL1 more recently) have been described as tumour suppressors, rhythmically controlling cell-cycle genes (16,20,21). For instance, downregulation of BMAL1 promotes tumour growth *in vivo* and accelerates cancer cell invasion *in vitro*, partially through its interplay with the tumour suppressor p53 pathway. In a recent genome-wide RNAi screen, downregulation of *Bmal1* allowed cells to bypass p53-dependent cell-cycle arrest (20). Our data show that expression of HPV16 E6/E7 oncogenes causes increased degradation of BMAL1 and disrupts circadian oscillations. Although extensive further work is needed to draw a definitive link, it is tempting to speculate that this disruption might affect the expression of downstream clock-controlled genes regulating the cell cycle (such as *Cyclin D1* and *Wee1*), contributing to tumorigenesis in HPV-related cancers.

Together, our results indicate UBE3A, an E3 ubiquitin ligase previously implicated in HPV-tumorigenesis and Angelman Syndrome, as a conserved and integral part of the circadian clock feedback loop. These findings not only reveal a *de novo* key regulator for the circadian transcription factor BMAL1 but also offer a new mechanistic link between cell transformation, intrinsic clock disruption and cancer.

## SUPPLEMENTARY DATA

Supplementary Data are available at NAR Online.

SUPPLEMENTARY DATA
