# Supplementary material for: The E3 ubiquitin ligase UBE3A is an integral component of the molecular circadian clock through regulating the BMAL1 transcription factor
Source: Nucleic Acids Res. 2014 Apr 11;42(9):5765–75. doi: 10.1093/nar/gku225 (PMC4027211; doi:10.1093/nar/gku225)
Supplement: SUPPLEMENTARY DATA [file supp_42_9_5765__index.html]

The E3 ubiquitin ligase UBE3A is an integral component of the molecular circadian clock through regulating the BMAL1 transcription factor — The E3 ubiquitin ligase UBE3A is an integral component of the molecular circadian clock through regulating the BMAL1 transcription factor — SUPPLEMENTARY DATA 

# The E3 ubiquitin ligase UBE3A is an integral component of the molecular circadian clock through regulating the BMAL1 transcription factor

## SUPPLEMENTARY DATA

**Files in this Data Supplement:**

- Supplementary Data
