# Supplementary material for: The E3 ubiquitin ligase UBE3A is an integral component of the molecular circadian clock through regulating the BMAL1 transcription factor
Source: Nucleic Acids Res. 2014 Apr 11;42(9):5765–75. doi: 10.1093/nar/gku225 (PMC4027211; doi:10.1093/nar/gku225)
Supplement: SUPPLEMENTARY DATA [file supp_gku225_nar-03612-v-2013-File008.pdf]

SUPPLEMENTARY INFORMATION

Supplementary Figure legends

Figure S1: Top panel: PCR showing stable integration of E6/E7 into genomic DNA of E6/E7 transformed cells. Bottom panel: RT-PCR showing expression of two splice variants (*) in cDNA as confirmed by sequencing. Unmarked band is non-specific.

Figure S2: Immunoflurorescence of p53 in primary (top) and transformed cells (bottom). Left panels show p53 staining only. Right panels show p53 staining (red) with DAPI (blue) and tubulin (green) counterstains. Scale 20 µm.

Figure S3: Soft agar assay demonstrating anchorage independent growth of E6/E7 positive cells (100% formed colonies, bottom), while primary cells remained single (top). Scale 50 µm.

Figure S4: PER2::Luc rhythms in primary and transformed cells following a temperature synchronisation protocol as indicated. Rhythm dampened rapidly upon removal of the temperature cycles.

Figure S5: (A) No significant changes of REV-ERBα protein level between wild type primary (WT) and E6/E7 transformed cells. Densitometry was based on three independent experiments. (B) Left panel: Application of MG132 (5 µM) could restore BMAL1 levels in E6/E7 transformed cells to a comparable level to primary wild type cells. Densitometry was based on three independent experiments. Right panel: Effective inhibition of the proteasomal degradation by MG132 (5 µM) in HEK 293T cells. Anti-ubiquitin western blot was performed using total proteins.

Figure S6: GSK3β and phophorylated (Ser9) GSK3β protein level in primary WT cells and E6/E7 transformed cells. Representative of three independent experiments.

Figure S7: (A) Western blots showing knockdown efficiency of two UBE3A shRNA constructs on overexpressed mouse myc-UBE3A (left) and human HA-UBE3A in HEK 293T cells. GFP plasmids were included as control for transfection efficiency. (B) Western blot showing knockdown efficiency of SMARTPool RNAi construct against endogenous UBE3A in SW1353 human chondrosarcoma cell line. Note the increased BMAL1 levels following RNAi treatments. Scrambled siRNA was included as a negative control.

Figure S8: The effect of CLOCK/BMAL1 over-expression on *mAVP*::luc promoter activity in HEK 293 cells. Data (mean ± SEM) was normalized to β-galactosidase reporter gene and expressed relative to pCDNA3 control. **p<0.01; t test. Knockdown of *Ube3a* significantly reduced the ability of BMAL1/CLOCK to activate *mAVP*::luc. Representative result of three independent experiments.

Figure S9: mRNA expression profile of *Ube3a* in multiple mouse tissues using RT-PCR. All tissues were harvested between 10-11 am from C57BL/6 mice kept in 12hr/12 hr light dark cycle. Equal amount of total RNA was reverse transcribed and used for PCR analysis. Expression of *18S* rRNA was used as internal loading control. PCR products were confirmed by DNA sequencing. Note the relatively higher expression in hypothalamus, pituitary and SCN.

Fig. S1

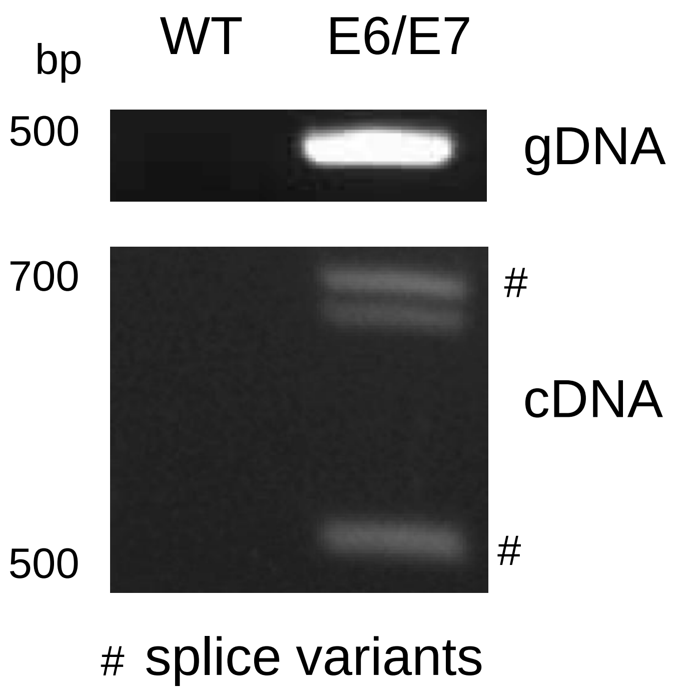

Fig. S2

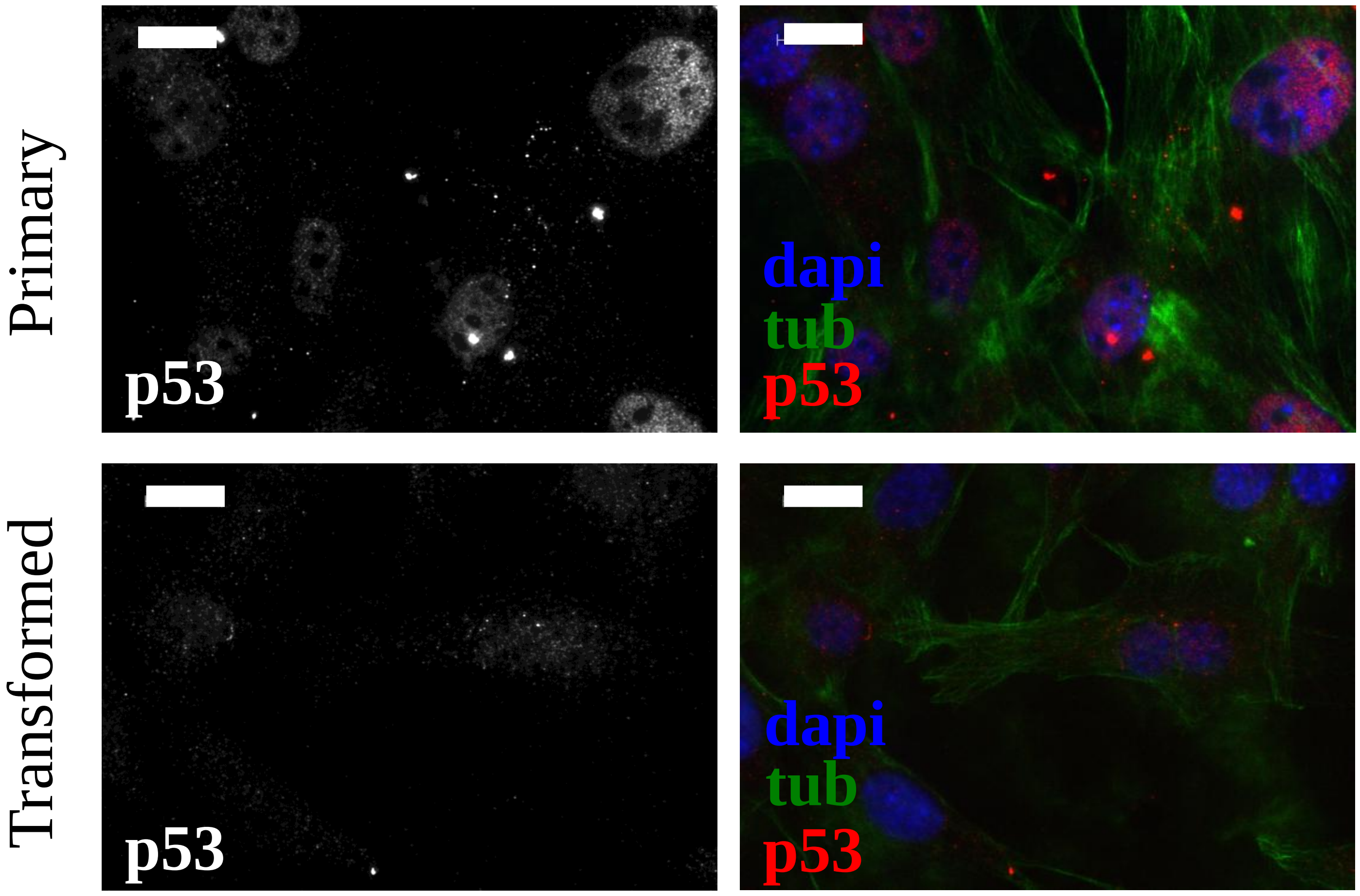

Scale: 20 μm

Fig. S3

WT

E6/E7

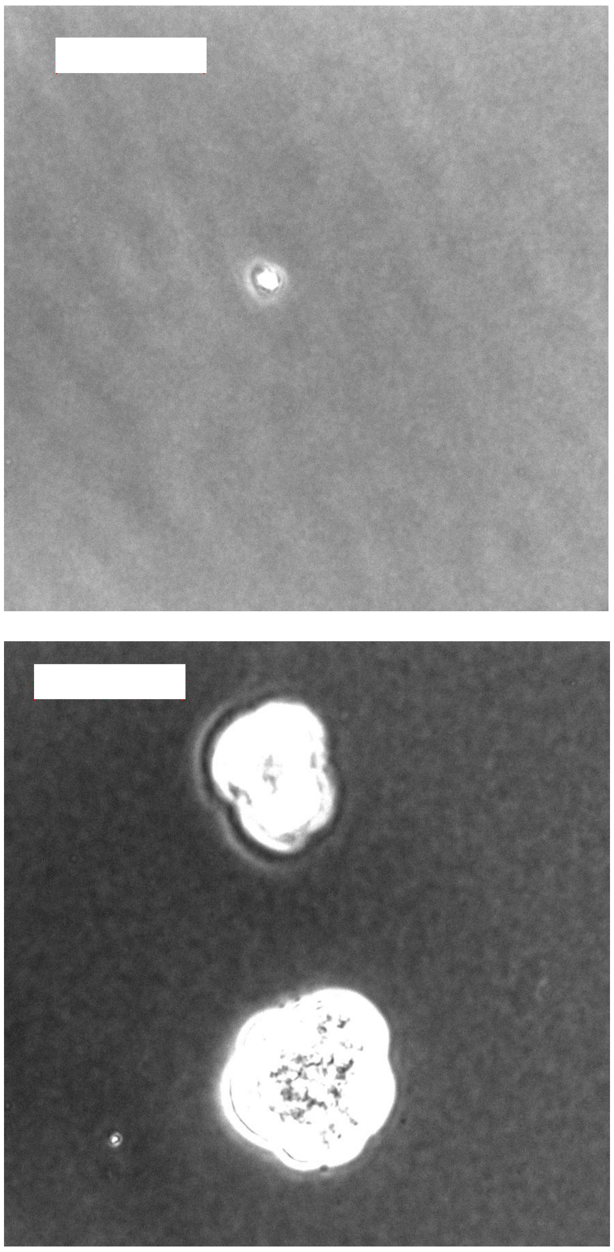

Fig. S4

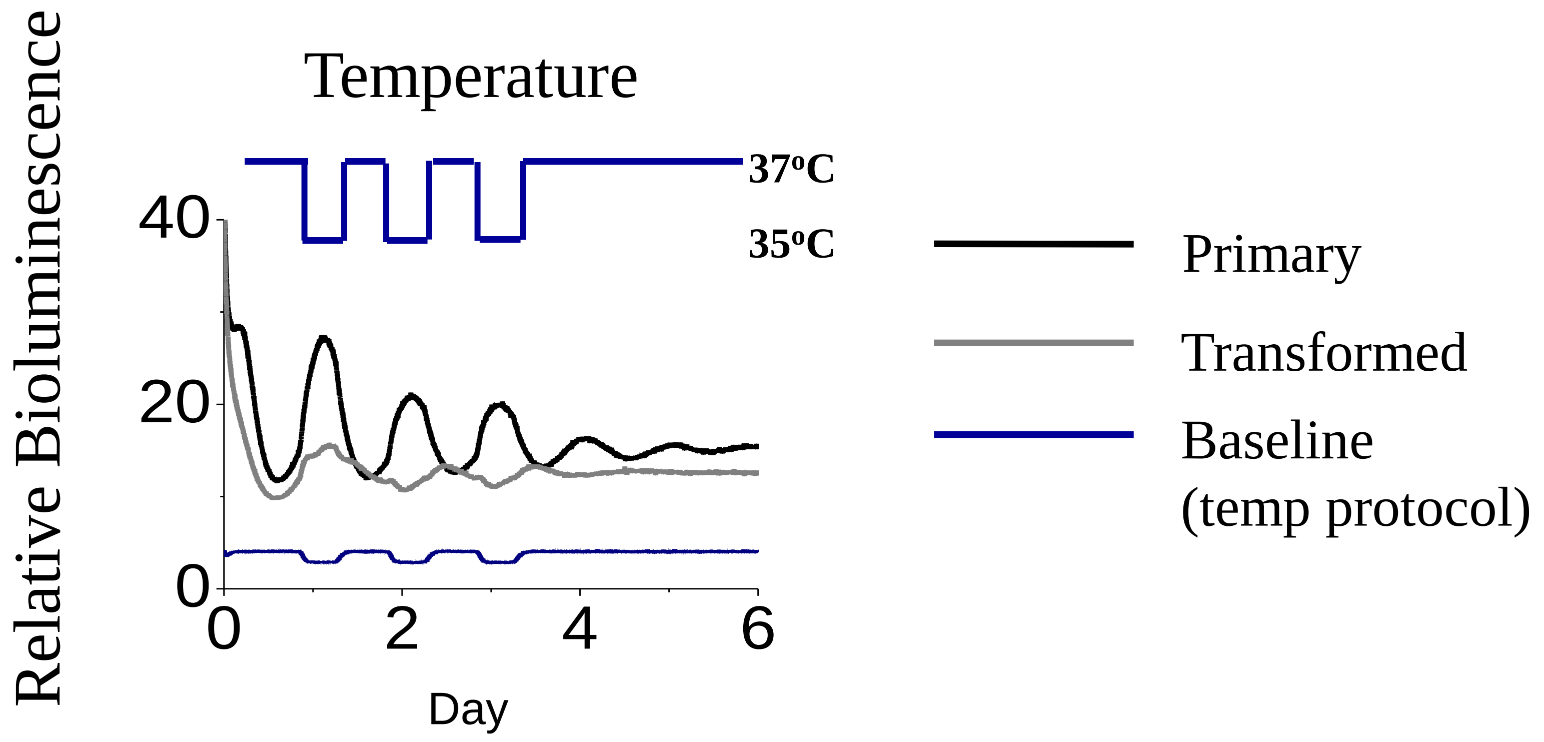

Temperature
37ºC
35ºC
Relative Bioluminescence
40
20
0
0
2
4
6
Day
Primary
Transformed
Baseline
(temp protocol)

Fig. S5

A

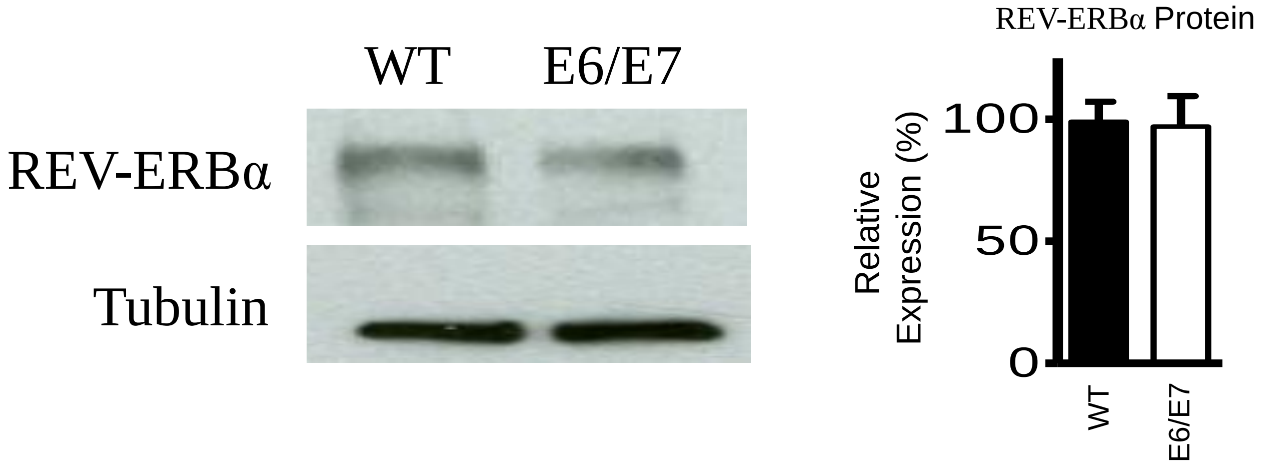
WT
E6/E7
REV-ERBα
Tubulin
REV-ERBα Protein
Relative Expression (%)
100
50
0
WT
E6/E7

B

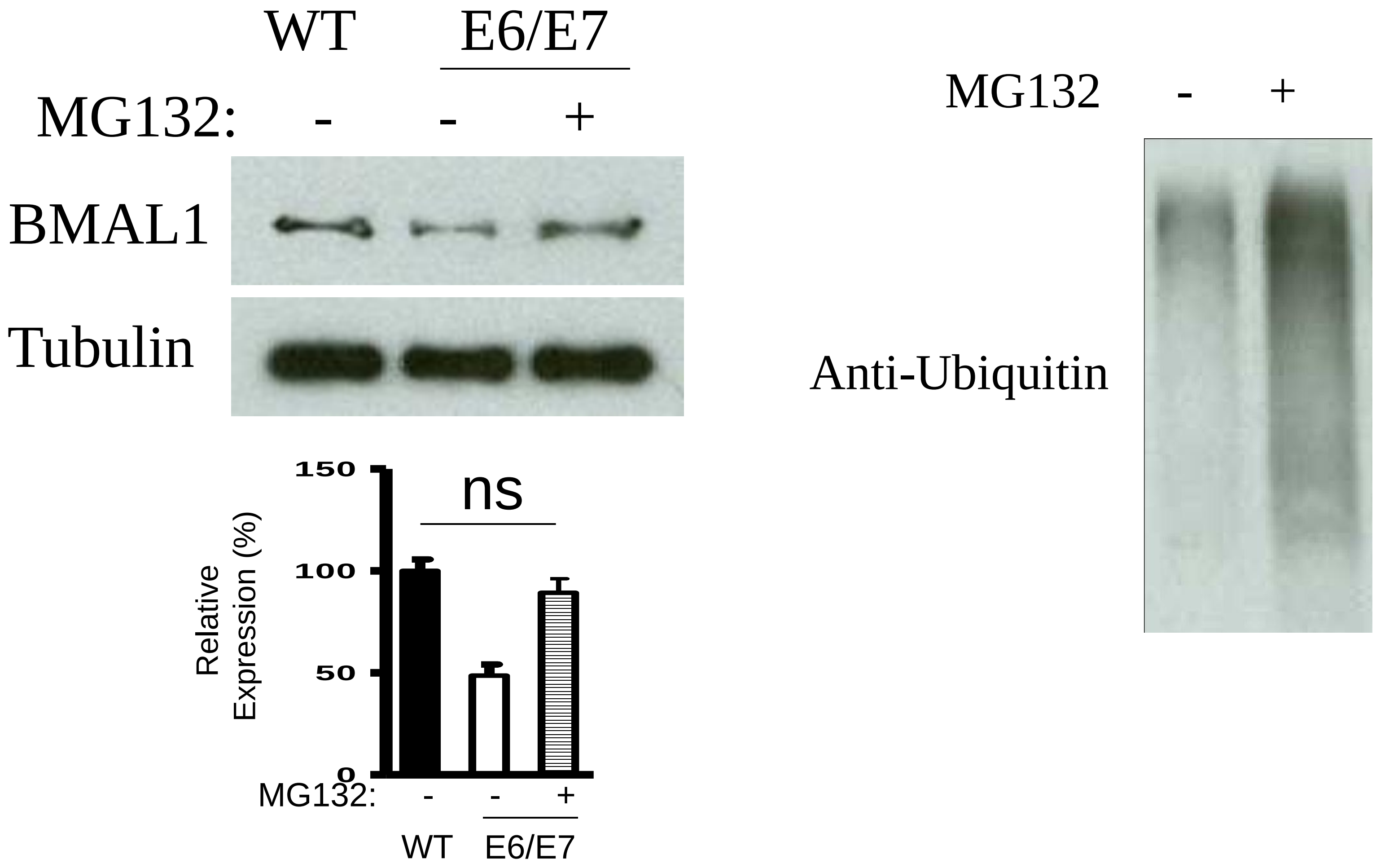
WT
E6/E7
MG132:
-
-
+
BMAL1
Tubulin
ns
150
100
50
0
Relative Expression (%)
MG132:
-
-
+
WT
E6/E7
MG132
-
+
Anti-Ubiquitin

Fig. S6

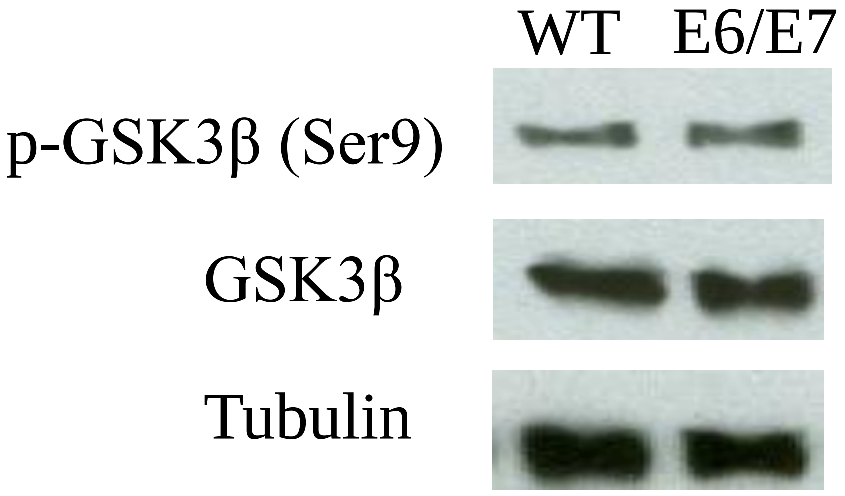

Fig. S7

A

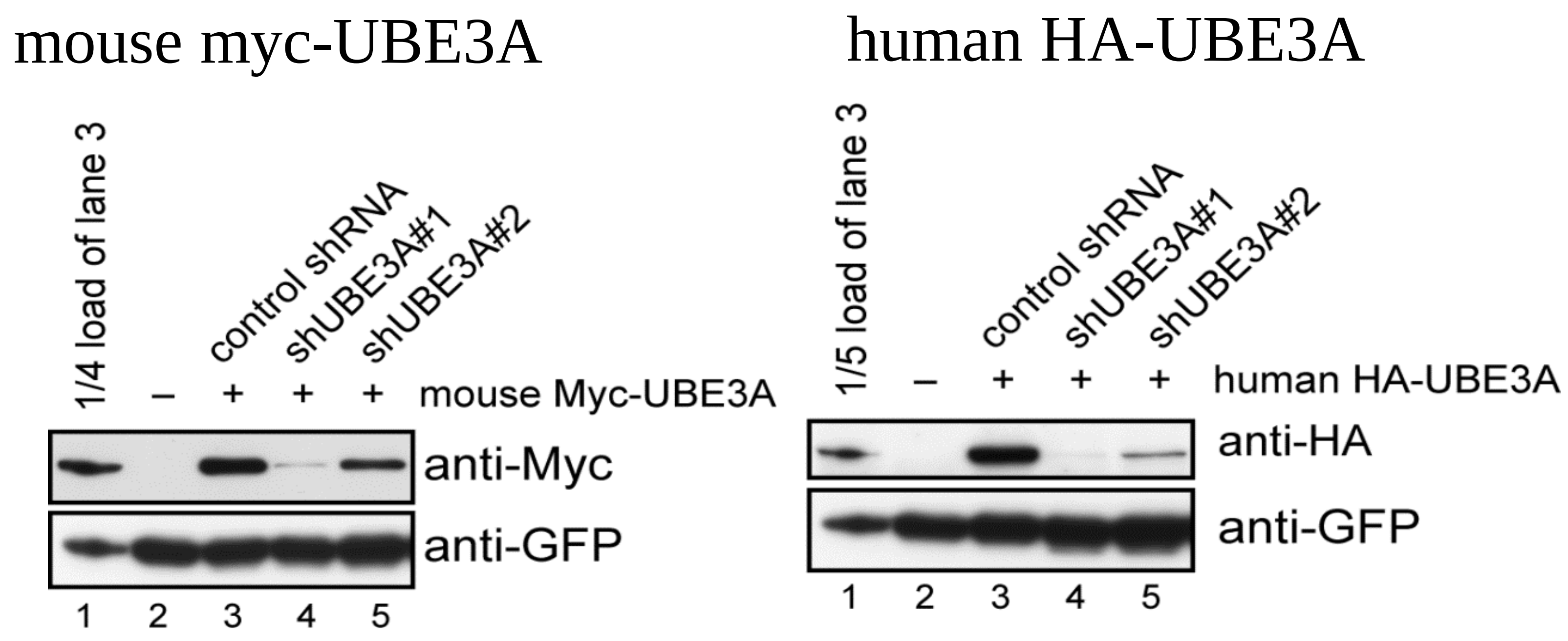
mouse myc-UBE3A
1/4 load of lane 3
control shRNA
shUBE3A#1
shUBE3A#2
– + + + mouse Myc-UBE3A
anti-Myc
anti-GFP
1 2 3 4 5
human HA-UBE3A
1/5 load of lane 3
control shRNA
shUBE3A#1
shUBE3A#2
– + + + human HA-UBE3A
anti-HA
anti-GFP
1 2 3 4 5

B

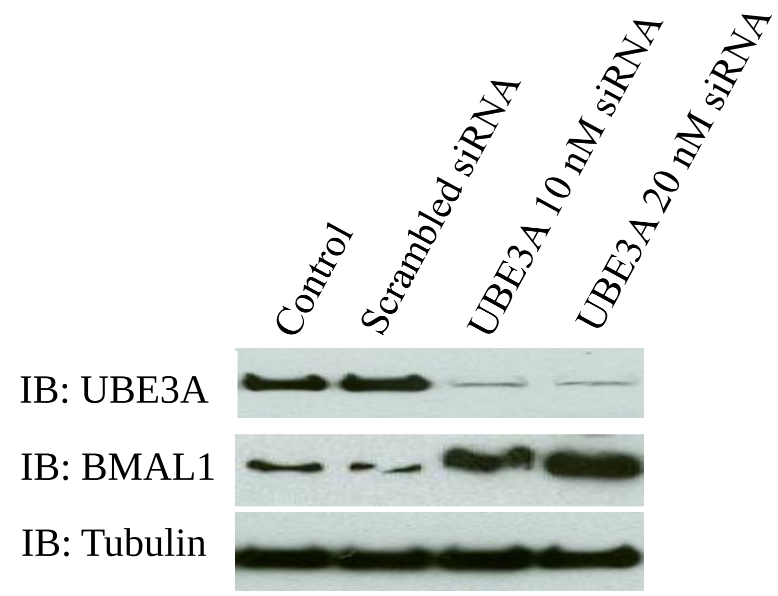
Control
Scrambled siRNA
UBE3A 10 nM siRNA
UBE3A 20 nM siRNA
IB: UBE3A
IB: BMAL1
IB: Tubulin

Fig. S8

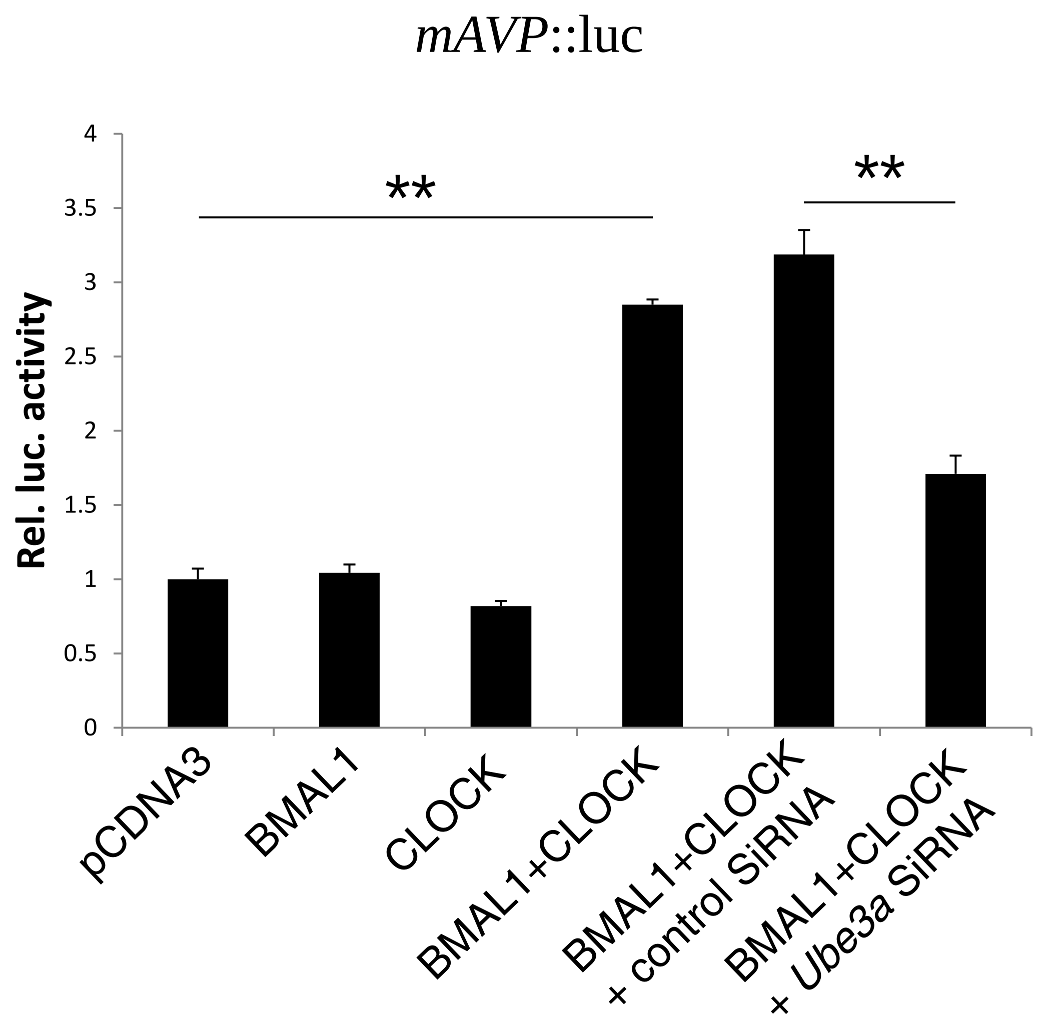
mAVP::luc
Rel. luc. activity
0
0.5
1
1.5
2
2.5
3
3.5
4
**
**
pCDNA3
BMAL1
CLOCK
BMAL1+CLOCK
BMAL1+CLOCK + control SiRNA
BMAL1+CLOCK + *Ube3a* SiRNA

Fig. S9

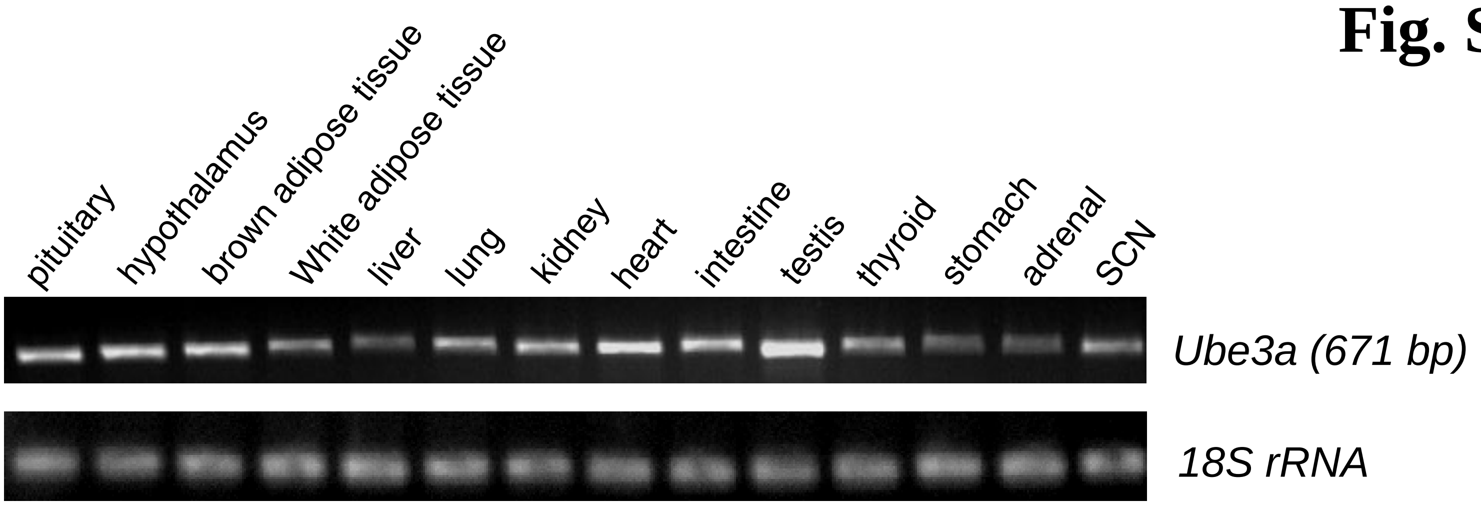
pituitary
hypothalamus
brown adipose tissue
White adipose tissue
liver
lung
kidney
heart
intestine
testis
thyroid
stomach
adrenal
SCN
Ube3a (671 bp)
18S rRNA
